# Inhibitors of *Mycobacterium tuberculosis* EgtD target both substrate binding sites to limit hercynine production

**DOI:** 10.1038/s41598-021-01526-6

**Published:** 2021-11-15

**Authors:** Thanuja D. Sudasinghe, Michael T. Banco, Donald R. Ronning

**Affiliations:** 1 Department of Pharmaceutical Sciences, University of Nebraska Medical Center, Omaha, NE 68198 USA; 2 Present Address: Biochemistry and Biophysics Center, National Heart, Lung, and Blood Institute, Bethesda, MD 20892-8012 USA

**Keywords:** X-ray crystallography, High-throughput screening, Transferases

## Abstract

Ergothioneine (EGT) is a low molecular weight histidine betaine essential in all domains of life but only synthesized by selected few organisms. Synthesis of EGT by *Mycobacterium tuberculosis* (*M. tb*) is critical for maintaining bioenergetic homeostasis and protecting the bacterium from alkylating agents, oxidative stress, and anti-tubercular drugs. EgtD, an *S*-adenosylmethionine-dependent methyltransferase (AdoMet), catalyzes the trimethylation of L-Histidine to initiate EGT biosynthesis and this reaction has been shown to be essential for EGT production in mycobacteria and for long-term infection of murine macrophages by *M. tb*. In this work, library screening and structure-guided strategies identified multiple classes of *M. tb* EgtD inhibitors that bind in various regions of the enzyme active site. X-ray crystal structures of EgtD-inhibitor complexes confirm that L-Histidine analogs bind solely to the L-Histidine binding site while drug-like inhibitors, such as TGX-221, and S-Glycyl-H-1152 span both the L-Histidine and AdoMet binding sites. These enzyme-inhibitor complexes provide detailed structural information of compound scaffolds useful for developing more potent inhibitors that could shorten Tuberculosis treatment regimens by weakening important bacterial defenses.

## Introduction

**Figure 1:**
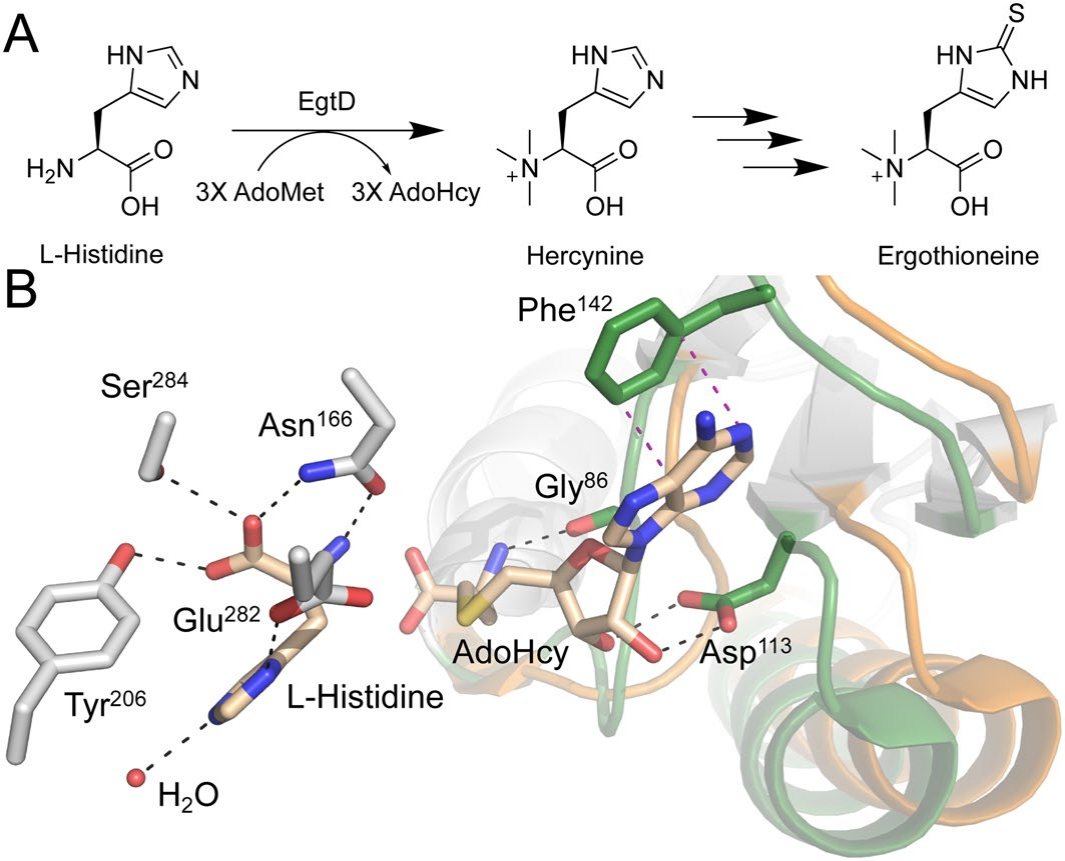
Synthesis of EGT and EgtD structure in actinobacteria. (**A**) Multi-step pathway in which L-Histidine is converted into EGT in *M. tb*. (**B**) Superposition of *M. smegmatis* EgtD in a complex with L-Histidine only (orange) and with both L-Histidine and AdoHcy (green) (PDB: 4uy6 and 4uy7). Residues interacting with L-Histidine are shown with gray carbons and those interacting with AdoMet are in green.

Ergothioneine (EGT) is a low molecular weight L-Histidine derivative required by all three domains of life^1^. Even though this ubiquitous molecule is found in all living organisms, the biosynthesis pathway of EGT has been found only in actinobacteria, cyanobacteria, methylobacteria, and some fungi (Fig. 1A)^2^.

A recent study has identified genes necessary for EGT biosynthesis in *Mycobacterium tuberculosis (M. tb)* where it plays a central role in maintaining bioenergetic homeostasis^3,4^. In addition, EGT defends *M. tb* against alkylating agents, heavy metals, oxidative stress, and anti-tuberculous drugs^4^. Due to these biologically important characteristics of EGT in *M. tb*, the enzymes essential for EGT biosynthesis represent intriguing targets for the development of new Tuberculosis (TB) treatments^2^. This is particularly relevant when considering the rising rate of drug-resistant TB and often poor response to the first-line TB drugs^5^. Novel inhibitors of EGT biosynthesis, when combined with existing antibiotics, is a potentially beneficial approach to treat both drug-sensitive and drug-resistant TB^6^.

By analyzing the genes encoding for these enzymes, a previous study has revealed that EgtB and EgtD are key enzymes in the biosynthesis of EGT^3^. Moreover, another study has found that the *egtD* gene, which encodes for EgtD in *M. tb*, is essential for EGT biosynthesis^2^. EgtD catalyzes the first step in this pathway by trimethylation of L-Histidine in an AdoMet-dependent reaction leading to the production of L-Hercynine (*N*(a), *N*(a), *N*(a)-trimethyl-L-histidine)^1^.

Multiple X-ray crystal structures of *Mycolicibacterium smegmatis* EgtD show that EgtD possesses a Histidine binding domain and a Rossmann-fold domain, which affords ordered substrate binding of L-Histidine and AdoMet, respectively^1,7^. These structures exhibit only minor side chain rotamer shifts in EgtD when bound to L-Histidine. Interestingly, the loop composed of residues 84–90 changes in response to AdoHcy binding and presumptively AdoMet binding from interactions with the methionyl moiety. Slight changes in neighboring α-helices support new interactions with the adenosyl moiety (Fig. 1B. Finally, the methyltransferase function of EgtD produces trimethylated L-Histidine (L-Hercynine) in a processive manner. Each of these important structural and functional features supports a strategy of bridging the L-Histidine and AdoMet binding sites for future inhibitor development^8^.

With the goal of ultimately developing EgtD inhibitors that engage both the L-Histidine and AdoMet binding sites simultaneously, this study describes the results of screening Histidine-/Histamine derivatives and two small-molecule libraries of drug-like molecules that bind the L-Histidine and AdoMet binding site of *M. tb* encoded EgtD. X-ray crystallographic evaluation of these EgtD-inhibitor complexes confirmed the value of this strategy and identified inhibitors able to bridge the substrate binding sites.

## Results

### Evaluation of L-Histidine-like inhibitors of EgtD

**Figure 2:**
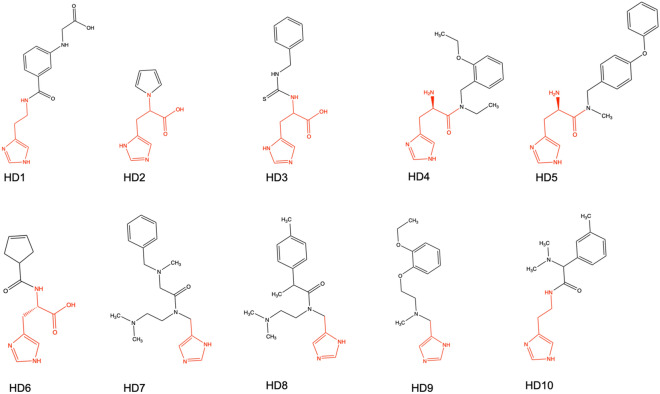
Histidine/Histamine derivatives library. Red color represents histidine/histamine mimicking moiety, and black represents the different functional groups tested. HD2 and HD3 are racemic mixtures.

A library of commercially available histidine and histamine derivatives were tested against EgtD to identify inhibitors that target the histidine binding site. Based on the previously solved EgtD crystal structures, there is limited space to accommodate the α-amino moiety of the bound L-Histidine. Therefore, different lengths of α-amino group linkers were tested as connecting linkers of L-Histidine and AdoMet binding sites. Figure 2 shows the structures of the tested compounds. EC_50_ values were determined to evaluate the structure–activity relationship of these compounds.

### Dose–response analysis of histidine/histamine derivatives

**Figure 3:**
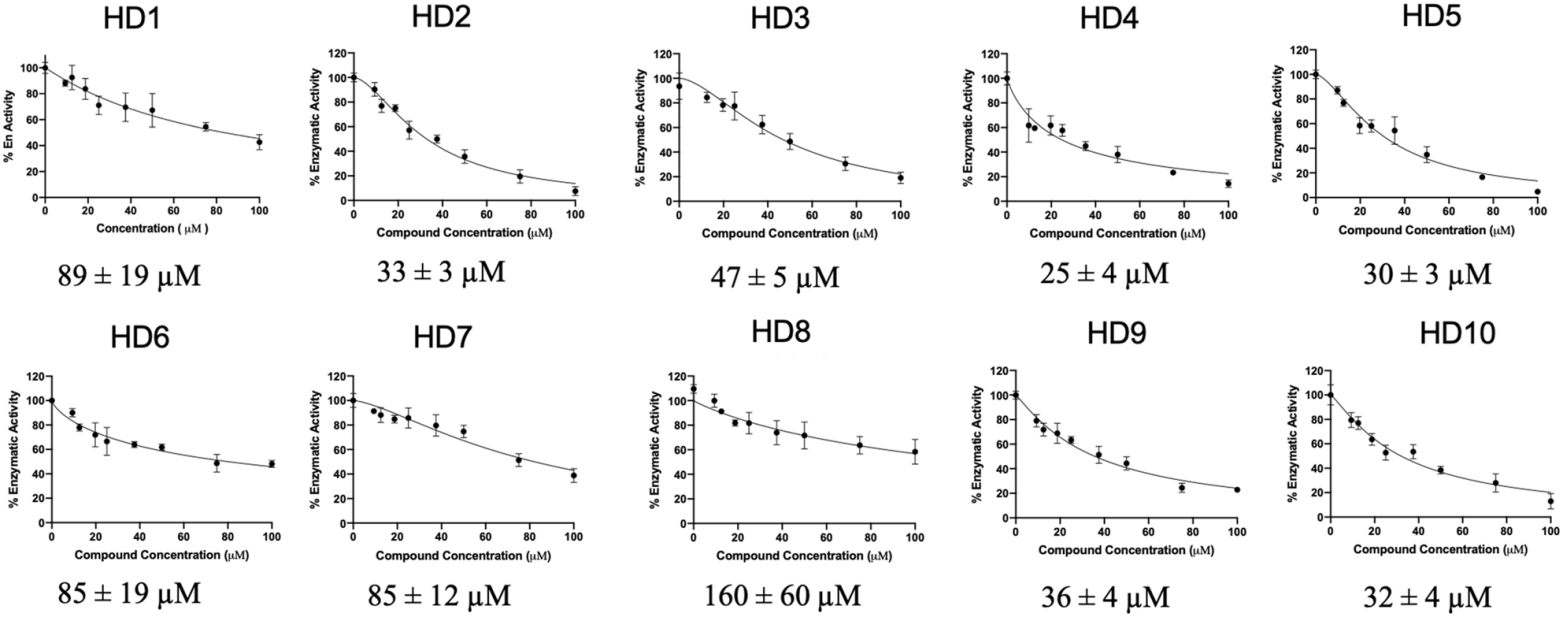
EC_50_ curves for histidine/histamine derivatives. The figure represents generated dose–response curves using a coupled fluorescence polarization assay. The EC_50_ values for the compounds are located below each plot and were calculated to the 95% confidence range.

Figure 3 shows the best fit EC_50_ values of the generated dose–response curves of histidine/histamine derivatives to a 95% confidence interval representing a one order of magnitude range of EC_50_ values spanning from 21 µM to 220 µM.

### Crystal structures of EgtD in combination with histidine/histamine derivatives and their interactions

EgtD protein was co-crystallized with each of the histidine/histamine derivatives to investigate the protein–ligand interactions and further characterize the structure–activity relationship of these compounds. The obtained crystals were subjected to X-ray diffraction experiments and X-ray crystal structures were obtained for *M. tb* EgtD with HD2, HD3, and HD6. X-ray diffraction and refinement statistics are shown in Table 1.

**Table 1:** X-ray diffraction data collection and refinement statistics.

Crystallographic Data	EgtD-HD2 (PDB: 7SCF)	EgtD-HD3 (PDB: 7SF5)	EgtD-HD6 (PDB: 7SEW)	EgtD-TGX221 (PDB: 7SEX)	EgtD-SGH (PDB: 7SEY)	EgtD-Imatinib (PDB: 7SF4)
Beamline/Facility	21-ID-D-APS	21-ID-D-APS	21-ID-D-APS	21-ID-D-APS	21-ID-D-APS	21-ID-D-APS
Wavelength (Å)	0.98	0.98	0.98	0.98	0.98	0.98
Space group	P2_1_2_1_2_1_	P2_1_2_1_2_1_	P4_1_2_1_2	P4_1_2_1_2	P4_1_2_1_2	P2_1_2_1_2_1_
Unit cell dimensions *a, b, c* (Å)	69.10, 71.57, 145.09	69.15, 70.28, 143.88	69.79, 69.79, 144.47	69.02, 69.02, 140.92	69.28, 69.28, 142.13	69.11, 71.26, 145.02
α, β, γ (°)	90, 90, 90	90, 90, 90	90, 90, 90	90, 90, 90	90, 90, 90	90, 90, 90
Resolution (Å)	50.95–2.67	49.85–2.52	49.35–1.72	49.31–2.20	39.11–2.39	40.00–2.81
No. reflections unique	21,089 (2068)	24,381 (2384)	31,266 (1383)	17,958 (1712)	214,149 (6673)	17,545 (1587)
Completeness (%)	99.84 (99.18)	99.86 (99.62)	80.57 (36.20)	99.78 (98.33)	88.10 (53.91)	96.77 (87.56)
R merge	0.10 (0.45)	0.10 (0.62)	0.09 (0.35)	0.08 (0.57)	0.15 (1.93)	0.15 (0.68)
CC 1/2	0.96 (0.44)	0.98 (0.55)	0.99 (0.45)	0.95 (0.67)	0.99 (0.63)	0.95 (0.72)
**Refinement**						
Resolution (Å)	50.95–2.67	49.85–2.52	49.35–1.72	49.31–2.20	39.11–2.39	40–2.81
No. Reflections (Unique)	21,067	24,352	31,250	17,957	12,659	17,457
R_work_ /R_free_	0.21/0.28	0.20/0.26	0.19/0.23	0.17/0.22	0.22/0.28	0.21/0.26
**No. atoms**						
Protein	4974	4974	2492	2493	2493	4985
Water	12	99	296	51	16	156
**B-factors**						
Protein	30.00	35.50	23.13	42.31	57.73	32.77
Water	26.00	35.20	34.81	45.50	56.73	37.19
**R.m.s. deviations**						
Bond lengths (Å)	0.01	0.01	0.01	0.01	0.01	0.01
Bond angles (°)	1.11	1.54	0.97	0.88	1.00	1.03

**Figure 4:**
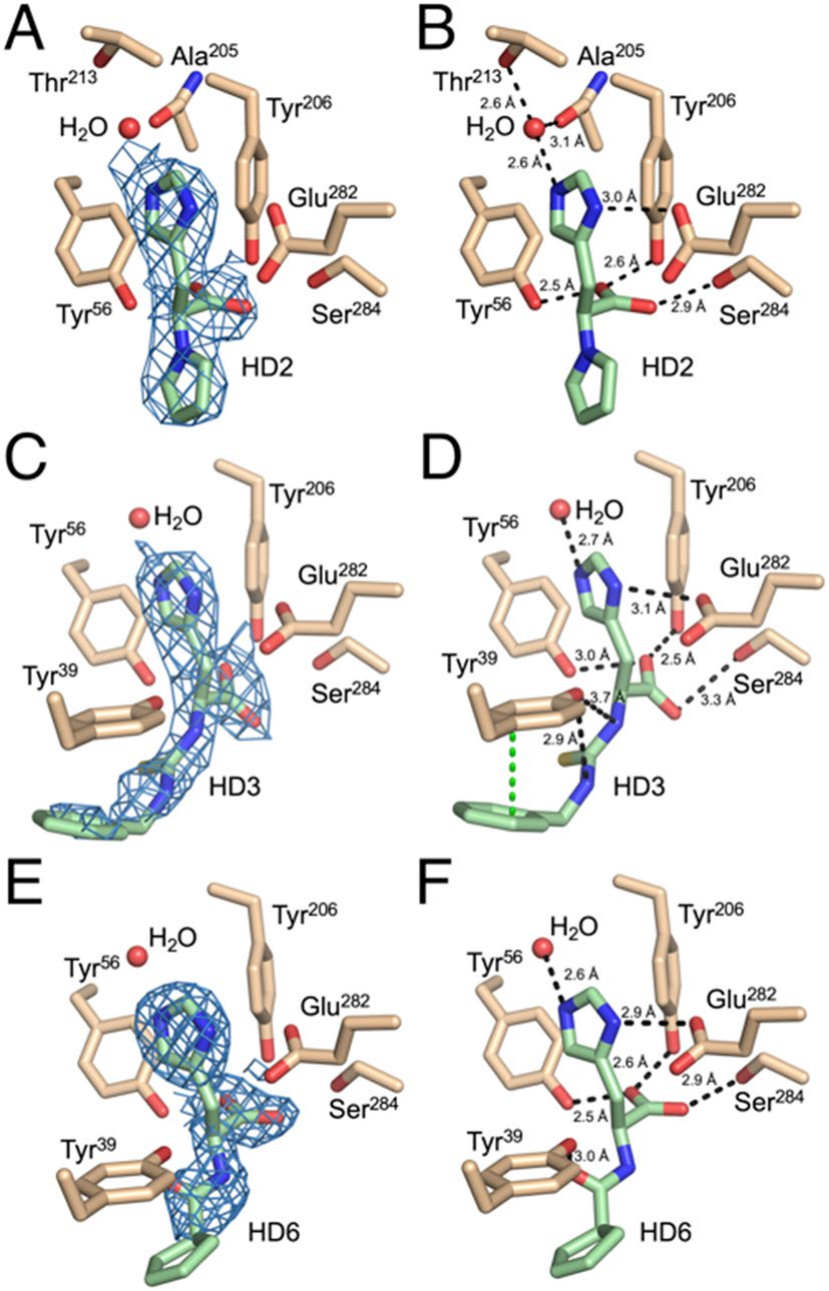
EgtD-HD2, EgtD-HD3 and EgtD-HD6 structures. (**A**, **C**, **E**) Composite omit 2Fo–Fc maps (blue) for Histidine/Histamine derivatives contoured to 1.0 σ. (**B**, **D**, **F**) Polar and π-π interactions of derivatives with the L-Histidine binding site. The ligands are colored in green (C: green, O: red, N: blue, and S: yellow), and the binding site residues are tan colored (C: tan, O: red, and N: blue).

As shown in Fig. 4, HD2 forms primarily polar interactions with the histidine binding site residues of EgtD. Specifically, HD2 forms a hydrogen-bonded interaction between an ordered water molecule, positioned by the Thr^213^ side chain and the backbone carbonyl of Ala^205^, and the basic nitrogen atom of the imidazole moiety. As with L-Histidine in published EgtD structures, the carboxylic group in HD2 forms hydrogen-bonded interactions with Tyr^206^, Ser^284^, and Tyr^56^. This compound is commercially available as a racemic mixture of L- and D- HD2 enantiomers. Based on the interactions observed in the EgtD complex with L-Histidine, it is reasonable to expect the L-enantiomer to be the dominant ligand. In this case, the 2.7 Å resolution of the EgtD-HD2 complex X-ray crystal structure is not sufficient to unambiguously define the stereochemistry of the bound inhibitor. The pyrrole moiety of HD2 forms a 3.6 Å van der Waals interaction with the α-carbon of Thr^163^ and a 3.3 Å van der Waals interaction with the side chain of Asn^166^.

The chemical structure of HD6 is very similar to that of HD2, so the interactions between EgtD and HD6 are nearly identical to those with HD2. The only difference observed is a rotamer change in the side chain of Glu^282^ resulting in slight reorientation of the carboxylate moiety. However, the presence of a hydrogen atom on the α-amino moiety of HD6 now affords a bidentate interaction between Asn^166^ and HD6, which is more similar to the interaction between EgtD and the L-Histidine substrate.

The interactions promoting binding of the HD3 L-Histidine moiety resemble those described for HD6 since both possess an α-nitrogen capable of donating a hydrogen bond to the side chain of Asn^166^. The benzylthiourea moiety of HD3 forms primarily non-specific van der Waals interactions with the side chains of Phe^47^ and Thr^163^ at distances between 3.2 and 3.8 Å. However, a π-π interaction forms between the HD3 benzyl moiety and the side chain of Tyr^39^ at a distance of 4.5 Å, which is unlikely to contribute significantly to binding.

### Discovery of drug-like inhibitors of EgtD

**Figure 5:**
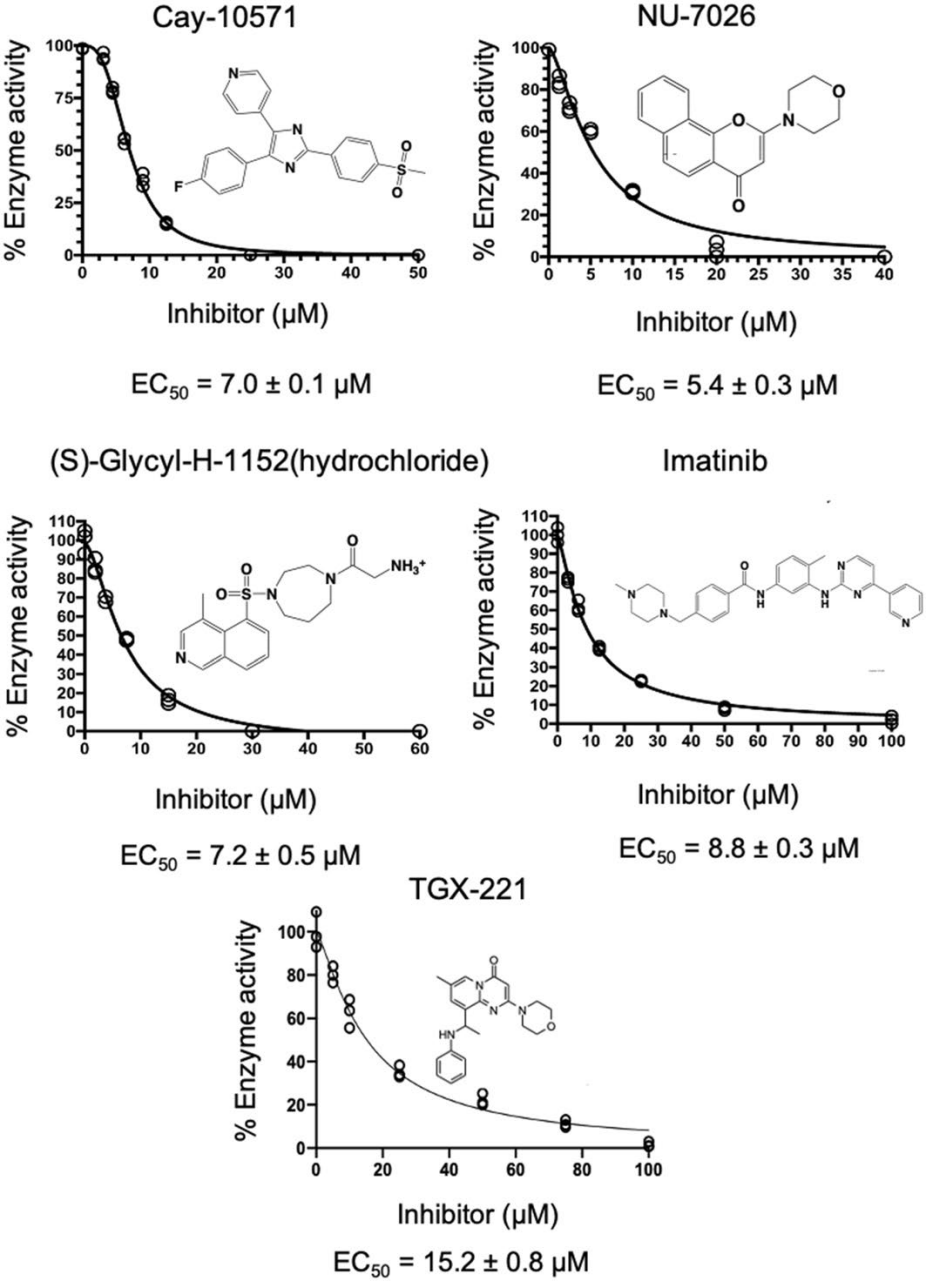
Dose–response curves for novel EgtD inhibitors. The figure shows the dose–response curves and calculated EC_50_ values for each inhibitor. The calculated EC_50_ values for the compounds are denoted below each plot.

To identify other classes of inhibitors and begin targeting the AdoMet binding site of EgtD, two small libraries, the NIH Clinical Collection (Charles River Laboratories) and a Kinase Inhibitor Library (Cayman Chemical) of drug-like compounds were screened using the same screening assay used to evaluate the L-Histidine derivatives. Five reproducible hits exhibiting a better than 3σ decrease in EgtD enzymatic activity were subjected to dose–response studies to determine EC_50_ values. Each hit from the group including TGX-221 (TGX221), Imatinib, (S)-Glycyl-H-1152 (SGH), Cay-10571, and NU-7026 exhibited an EC_50_ lower than 20 μM (Fig. 5).

**Figure 6:**
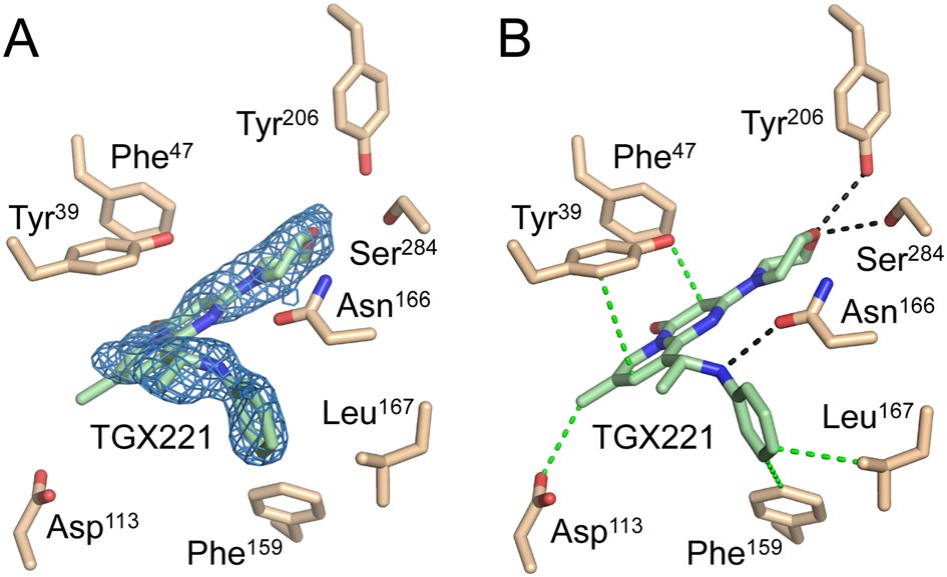
EgtD-TGX221 interactions. (**A**) The 2Fo-Fc omit map of the TGX221 ligand contoured at 1.0 σ. (**B**) The van der Waals interactions and polar interactions between the ligand and protein are shown in green and black, respectively.

To investigate the protein–ligand interactions of the identified inhibitors, a subset of these compounds was co-crystalized with EgtD, which included TGX221, Imatinib, and SGH. The EgtD-TGX221 complex X-ray crystal structure was determined to 2.2 Å resolution. As illustrated in Fig. 6, difference density in the initial maps correspond to bound TGX221 within the portion of the EgtD L-Histidine binding site known to accommodate the α-amino and α-carboxylate moieties of that substrate. However, the L-Histidine side chain sub-site is not filled by TGX221 but instead harbors a glycerol molecule.

This complex is further stabilized through the formation of hydrogen-bonded interactions between residues Asn^166^ and Ser^284^ and the aniline and morpholine moieties of TGX221, respectively. The aniline moiety of TGX221 also forms van der Waals interactions with the side chains of Phe^142^, Phe^159^, and Leu^167^. Additionally, π-π interactions between the TGX221 fused pyrido-pyrimidinone moiety and Tyr^39^ side chain at a distance of 4.0 Å support binding, while the 3.5 Å interaction between the side chain of Asp^113^ and the 7-methyl moiety elaborating the pyrido-pyrimidinone of TGX221 may be slightly energetically unfavorable due to the relative chemical incompatibility of the methyl and carboxylate moieties.

**Figure 7:**
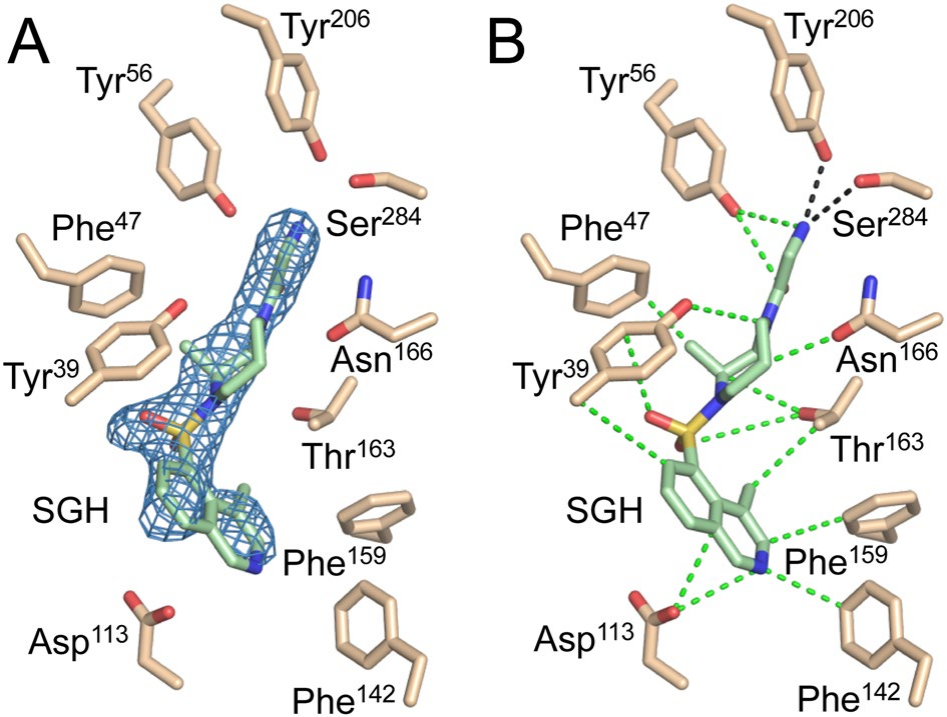
EgtD-SGH interactions. (**A**) The composite omit 2Fo-Fc map for the ligand contoured to 0.9 σ. (**B**) The various protein–ligand interactions that contribute to SGH binding are shown with van der Waals interactions in green dashed bonds and polar interactions with the Histidine binding site residues Tyr^206^ and Ser^284^ in black dashed bonds.

The 2.4 Å-resolution EgtD-SGH structure shows a clear overlap between the TGX221 and SGH binding sites (Fig. 7). In contrast to the interactions with TGX221, the interactions between *M. tb* EgtD and SGH are nearly all non-specific van der Waals interactions. Indeed, SGH does not enter the subsite that binds the L-Histidine side chain and the primary specific hydrogen-bonded interactions are formed by the terminal primary amine moiety of SGH with the side chains of Tyr^206^ and Ser^284^, which are responsible for binding the carboxylate moiety of L-Histidine. Additionally, Glu^282^ is forming an apparent ionic interaction with the terminal amine of SGH, which suggest that SGH may be represented by the protonated ammonium form. The rest of SGH occupies the volume of space reserved for binding the L-Histidine α-amino moiety and the AdoMet sulfonium moiety and then encroaches on the large volume of the adenosyl-binding subsite. However, SGH does not interact through specific polar interactions with the important EgtD residues such as Asn^166^ or Glu^282^. In the AdoMet binding site, SGH forms eight van der Waals interactions where two of these are likely destabilizing.

**Figure 8:**
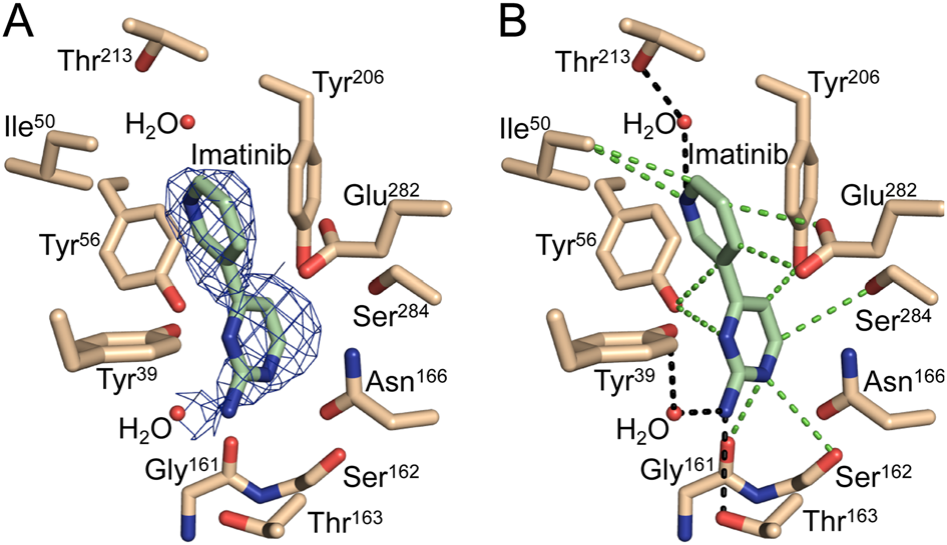
EgtD-Imatinib interactions. (**A**) 2Fo-Fc composite omit map for the imatinib pyridine-pyrimidine moiety at 1.3 σ and the interactions with the residues of the L-Histidine binding site that support complex formation. (**B**) Polar interactions are indicated with black dashed bonds and van der Waals interactions with green dashed bonds.

Imatinib is a tyrosine kinase inhibitor that exhibits synergy with first-line TB drugs in macrophage infectivity studies. The current hypothesis is that inhibition of human kinases by Imatinib affects the response to the *M. tb* infection. It was therefore expected that Imatinib would bind within the EgtD AdoMet binding site. However, as shown in Fig. 8, we instead observed that a portion of Imatinib, the terminal pyridine-pyrimidine rings, is bound within the EgtD L-Histidine binding site. Based on the density calculated from X-ray diffraction data extending to a resolution of 2.8 Å, attempts were also made to model the observed density using the piperazine moiety at the other terminus of the Imatinib molecule. However, the resulting steric hindrance by EgtD residues forming the active site prevented satisfactory fitting of Imatinib in that orientation.

The two rings of Imatinib modeled within the L-Histidine binding pocket are well-resolved. The remainder of Imatinib extends to the AdoMet binding site resulting in a lack of difference density representing those three heterocycles. The pyridine ring sits within the imidazole binding subsite and forms π-π interactions with Tyr^206^ and eleven close (shorter than 4.0 Å) van der Waals interactions with the surrounding hydrophobic residues. In addition, the nitrogen atom of the pyridine ring forms a hydrogen bond with the ordered water molecule analogous to the imidazole-water interaction observed in the L-Histidine derivative structures. The pyrimidine ring forms only three van der Waals interactions and three hydrogen bonded interactions with two ordered water molecules and the side chain of Thr^163^.

## Discussion

Previous studies have shown that the EgtD catalysis proceeds via processive trimethylation of L-Histidine^8^. Based on that study, L-Histidine binds first to the histidine binding site. Then, AdoMet binds and induces the positioning of the AdoMet methyl sulfonium moiety to within approximately 1.7 Å of the α-amino group of L-Histidine to afford efficient methyl transfer^8^. Subsequent release of the first AdoHcy product affords binding of an additional AdoMet molecule. This process repeats until the final production of Hercynine. The observed crystal structures of EgtD-Histidine-/Histamine-derivative complexes, consistent with the changes observed upon L-Histidine binding to EgtD, exhibit only minor variation in the side chain rotamers of residues forming the substrate binding sites. Along with the determined *M. smegmatis* EgtD crystal structures, this suggests a relatively long residence time of L-Histidine a the EgtD active site^1^. Therefore, it follows that L-Histidine-competitive analogs forming similar interactions with EgtD would bind with high affinity and selectivity. Further, by linking such EgtD inhibitors to compounds competitive with EgtD AdoMet binding, this would offer a unique set of compounds exhibiting limited cross-reactivity with human proteins.

The determined EC_50_ values show bimodal clustering of inhibitory potency with the best class being between 25 and 50 µM and a minority of compounds ranging from 85 to 160 µM. The measured inhibitory activity of these derivatives is on the same order of similar L-Histidine analogs tested against the *M. sm*egmatis-encoded EgtD suggesting that the conserved active site residues of the EgtD homologs are interacting with inhibitors in a consistent manner^1^. The differences in potency between the analogs reported here are readily rationalized by inspection of the presented X-ray crystal structures and comparison of those structures to subclasses of the HD compounds based on the identity and bulk of the chemical linker between the imidazole moiety and the remainder of the compound. First, HD2, HD3, and HD6 most closely resemble a Histidine amino acid as they all possess α-amino and α-carboxyl moieties, but the α-amino group is elaborated with a variety of chemical linkages. As expected, these binding modes closely resemble that of L-Histidine with the minor exception of the α-carboxylate moiety of HD3. Possibly due to the added bulk of the thiourea coupling to the benzyl moiety, the ψ dihedral connecting the α-carboxylate to the α-carbon has rotated by 30°, which results in the lengthening of two hydrogen-bonded interactions with Tyr^56^ and Ser^284^ and the minor decrease in the inhibitory activity of HD3 with respect to HD2. The reason for the 1.5–2.5-fold decrease in the HD6 inhibitory activity versus HD2 and HD3 is less clear but is likely derived from steric hindrance between the cyclopentene and the narrow constriction separating the Histidine and AdoMet binding sites. In contrast, HD3 possesses two freely rotating bonds between the aryl moiety and the thiourea linker, which suggests it is more easily accommodated by this region of EgtD.

**Figure 9:**
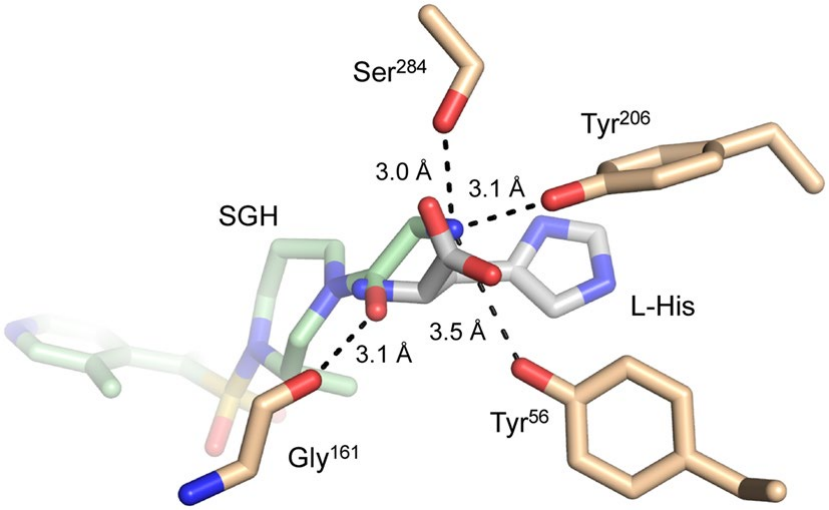
Superposition of *M. tb* EgtD-SGH (green) and EgtD-L-Histidine (gray) structures. Side chains forming the α-carboxylate binding site (tan carbons) readily accommodate a primary amine and amide linkage in the SGH complex. Binding modes similar to this region of SGH are likely in the HD4 and HD5 complexes.

The knowledge obtained from the described EgtD/HD complex structures forms a basis for inferring the mode of binding of the other tested HD derivatives. HD4 and HD5 exhibited the best inhibitory activity against *M. tb* EgtD with EC_50_ values of 25 ± 4 µM and 30 ± 3 µM, respectively. HD4 and HD5 differ from the other Histidine analogs in that they possess a primary α-amine and an amide linkage to the remaining chemical moieties. Therefore, HD4 and HD5 somewhat resemble a dipeptide. While crystal structures of an EgtD/HD4 or HD5 complex are not yet available, it is reasonable to expect a similar EgtD binding mode to that observed in the determined HD complex structures. However, HD4 and HD5 both lack an α-carboxylate moiety capable of forming the tetradentate interactions observed for L-Histidine. Therefore, the most likely binding mode of HD4 and HD5 in the EgtD Histidine binding site requires a rotation of the χ1 bond of the Histidine moiety to place the α-amino moiety in the EgtD carboxylate binding site to interact with hydrogen bond acceptors Tyr^206^ and Ser^284^. Indeed, this proposed interaction is like that observed in the EgtD/SGH complex (Fig. 9). This χ1 bond rotation would also allow the amide coupled moieties of HD4 and HD5 to bind within the EgtD AdoMet binding site. However, testing of mono- or di-methylation of the a-amino moiety, similar to those Histidine analogs reported by Vit et al*.* would address this question and could potentially lead to higher potency inhibitors of this class^7^.

Concerning the HD7, HD8, and HD9 derivatives, HD7 and HD8 both possess tertiary amines with *N*,*N*-dimethyl ethylamine branch only one carbon removed from the imidazole moiety. HD8 has an additional methyl branch for added bulk. These are likely incapable of engaging the EgtD Histidine binding site in a manner consistent with that seen for the other HD compounds. In contrast, the ternary amine of HD9 has only a methyl group, which is easily accommodated in the a-carboxylate binding site. The inherent flexibility connecting the two ring systems in HD9, although not desirable for the development of highly potent drugs, seems to afford improved binding in HD9 when compared to HD7 and HD8.

HD1 and HD10 are Histamine analogs linked by an amide to an aryl, HD1, or a branched methylene linker to an aryl group, HD10. The amide-linked moieties in both these compounds are relatively bulky and both possess an amine group that may harbor a positive charge. Without the insight offered by an X-ray crystal structure of either EgtD complex, it seems most likely that the carboxylic acid on HD1 is forming an energetically unfavorable interaction with residues in the AdoMet binding site.

To move beyond the Histidine binding site and better engage the AdoMet binding site, ATP-competitive kinase inhibitors were tested due to the obvious structural similarities between ATP and AdoMet. The identification of Imatinib as an inhibitor of *M. tb* EgtD is intriguing. While Imatinib is a well-known inhibitor for various tyrosine kinases such as ABL1, ABL2, KIT, and PDGFR and has been used to treat chronic myelogenous leukemia and solid tumors, little is known about how it can affect intracellular bacterial infections^9^. Previous studies using Imatinib have shown a decrease in the intracellular replication of *M. tb* and *Mycobacterium marinum* (*M. marinum*) in macrophage cells and infected mice^10–12^. The current hypothesis is that Imatinib disrupts *M. tb* cellular entry^10–12^. However, treatment of *M. marinum*-infected macrophage cells with Imatinib and Rifampicin exhibited a synergistic effect^10^. As mentioned previously, knockouts of EgtD in *M. tb* are more susceptible to various anti-tubercular drugs, including Rifampicin^4^. Based on these results, it is intriguing to consider that *M. tb* EgtD is a biological target for Imatinib and that inhibition of EGT production during an infection is the reason for the observed drug synergy.

**Figure 10:**
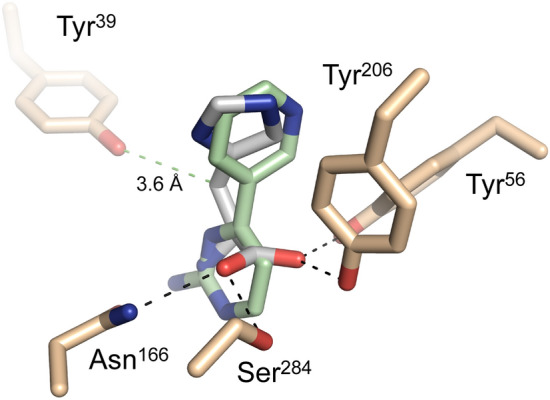
Superposition of *M. tb* EgtD-Imatinib (green) and EgtD-L-Histidine (gray) structures. Highlighted are the interaction differences between the L-Histidine and Imatinib structures. In the L-Histidine complex Tyr^56^, Tyr^206^, Ser^284^, and Asn^166^ of EgtD. Tyr^39^ forms a van der Waals interaction with the α-carbon of L-Histidine that is lacking in the Imatinib complex.

As shown in Fig. 8, the pyridine-pyrimidine rings of Imatinib are positioned in the histidine binding pocket by an extensive network of interactions. Superimposing the Imatinib complex with the *M. smegmatis* EgtD-L-Histidine complex illustrates distinct modes of binding site engagement (Fig. 10). Where L-Histidine interacts with EgtD primarily through polar interactions, Imatinib interacts through almost exclusively van der Waals interactions. This structural comparison can be used to develop a library of second-generation EgtD inhibitors that are L-Histidine competitive with the selectivity of L-Histidine but possessing additional van der Waals interactions reflective of the Imatinib binding mode to support more potent binding.

**Figure 11:**
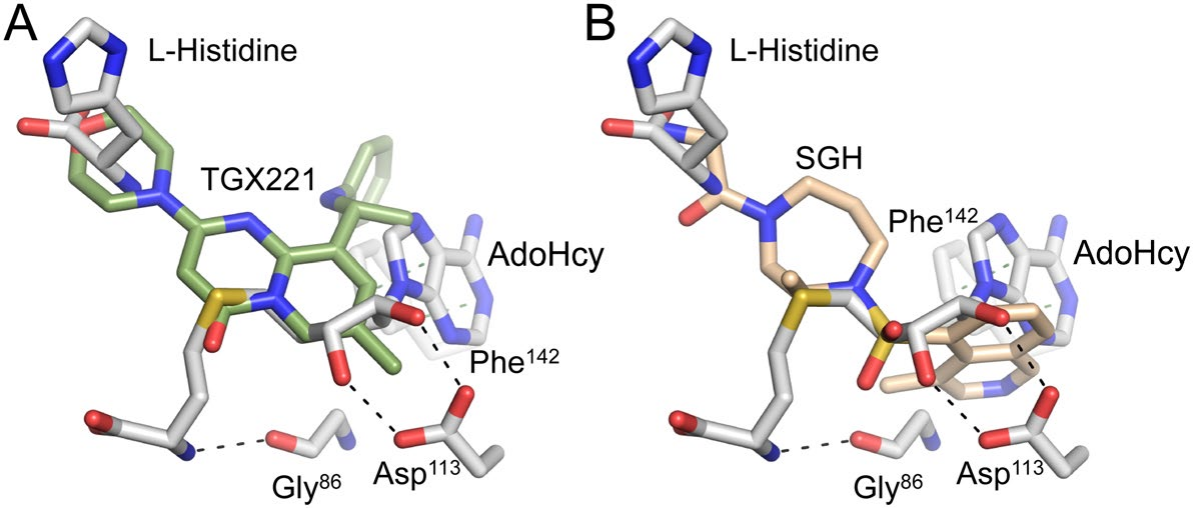
Required interactions for EgtD active site closing. (**A**) Super position of *M. tb* EgtD-TGX221 complex (green carbons) with closed *M. smegmatis* EgtD complex (gray carbons) (**B**) Super position of *M. tb* EgtD-SGH complex (tan carbons) with closed *M. smegmatis* EgtD complex (gray). The indicated polar (black dashed bonds) and non-polar interactions (green dashed bonds) are required for EgtD active site closing. Each of the interactions are lacking in both the TGX221 and SGH inhibitor complexes.

As previously stated, the original rationale for testing kinase inhibitors was to identify compounds capable of engaging the EgtD AdoMet binding site with the ultimate goal of developing inhibitors that stimulate closing of the EgtD active site as observed for the EgtD-L-Histidine-AdoHcy ternary complex (Fig. 1). The presented X-ray crystal structures of the EgtD-inhibitor complexes with TGX221 and SGH clearly show that these inhibitors bridge the Histidine binding site and a portion of the AdoMet binding site but are unable to stimulate closing of the EgtD active site as EgtD active site closing relies on polar interactions with the backbone carbonyl of Gly^86^ and the side chain of Asp^113^ as well as a π-π interaction between the adenyl moiety of AdoMet and the Phe^142^ side chain (Fig. 11). Any new TGX221 and SGH derivatives would benefit by supporting these interactions.

## Conclusion

This study reports the discovery of *M. tb* EgtD inhibitors that target the Histidine binding site as well as bridge the Histidine and AdoMet binding sites. Based on the inhibitory and structural studies of EgtD-Histidine-/Histamine-derivative complexes, ten target compounds were identified and evaluated in dose–response and X-ray crystallography. Correlation of the structural information from L-Histidine analogs and Imatinib bound to EgtD highlights intriguing inhibitor possibilities for bicyclic systems possessing both high potency and selectivity. In addition, the discovery of the TGX221 and SGH inhibitory activities and the ability of these compounds to bridge portions of the L-Histidine and AdoMet binding sites will lead directly to compounds highly specific to the EgtD due to the unique conjunction of the two substrate-binding sites and therefore avoid inhibition of other AdoMet-dependent enzymes. Additionally, it can be expected that such inhibitors capable of fully closing the EgtD active site would exhibit significantly higher potency than those described here and be expected to exhibit a profound decrease of *M. tb* EGT production. Such efficacy would thereby increase the bacterial susceptibility to both the human immune response and anti-tubercular drugs. Both outcomes would shorten the duration of TB treatments.

## Methods

### Molecular cloning, expression, and purification of EgtD

A gene encoding the *M. tb* EgtD protein was codon-optimized for expression in *E. coli* and inserted into a pET32-derived expression vector. This plasmid encodes a recombinant EgtD enzyme possessing a rhinovirus 3C protease-cleavable N-terminal thioredoxin and polyhistidine tag. After confirming the DNA sequence, the engineered plasmid was used to transform high-efficiency chemically competent T7 cells for protein expression. Cells were cultured at 37 °C in LB medium containing carbenicillin. Once the density of the culture reached OD_600 nm_ = 0.6, the temperature of the shaking incubator was decreased to 16 °C. When the culture was cooled to 16 °C, 1 mM isopropyl β-D-1-thiogalactopyranoside was added to induce protein production. After 24 h of induction, the cells were harvested by centrifugation at 3724 × *g* at 4 °C. The cell pellets were resuspended in lysis buffer containing 50 mM HEPES (pH 7.5), 300 mM NaCl and 10% glycerol. Then the cell suspension was treated with DNase I and Lysozyme and incubated on ice for 30 min. Following sonication, the cell lysate was centrifuged at 18,515 × *g* and 4 °C for 40 min to pellet the cell debris. The clarified supernatant was loaded onto a 5 mL cobalt affinity column (Cytiva) pre-equilibrated with 30 mL of lysis buffer. The column was then washed with 15 column volumes of lysis buffer to remove unbound and non-specifically bound proteins. His-tagged protein was then eluted using an Imidazole gradient from 0 to 150 mM. Rhinovirus 3C protease was added to the eluted protein to cleave the affinity tag and the sample dialyzed for 16 h at 4 °C against lysis buffer. The dialyzed protein solution was applied to a 5 mL cobalt column pre-equilibrated with lysis buffer and the cleaved recombinant EgtD protein was collected in the flow through. Purified EgtD protein was concentrated and employed for enzymatic assays and crystallization experiments.

### EgtD inhibitor library screening

Screening of recombinant EgtD methyltransferase activity was performed against the Cayman Chemical Kinase Inhibitor library and the histidine analogs followed the procedure previously described^13^. Briefly, stock solutions of inhibitors at a concentration of 10 mM and dissolved in 100% DMSO were added to the well of a 384-black well plate. Each well contained a 48.5 µL assay mixture containing the following: 51.5 nM HpMTAN-D198N, 11.6 nM AdoHcy-TAMRA, 11.1 nM EgtD, 773 µM L-Histidine, and 51.5 mM TRIS pH 8.0. The addition of 0.5 µL of each tested compound resulted in a final inhibitor concentration of 100 µM and 1% DMSO. Each reaction was then initiated by the addition of 1 µL of a 500 nM AdoMet stock solution. All reactions for were monitored continuously on a Biotek Synergy H4 plate reader with excitation and emission wavelengths of 545 nm and 580 nm, respectively. An analogous assay with indicated concentrations of the hit compound was performed to assess the dose–response. After subtracting the background signal, the percent enzymatic activity (EC_50_) for each reaction was calculated using the following equation where V’ represents the inhibited rate, and V’o is the uninhibited rate of the DMSO control.

$$\% {\text{Enzymatic}}\;{\text{activity}} = \left( {{\text{V}}^{{\prime }} /{\text{V}}_{0}^{{\prime }} } \right)*{1}00$$

Then the % enzymatic activity was plotted as a function of inhibitor concentration. The data were fitted to the “[Inhibitor] vs. normalized response–Variable slope” using GraphPad Prism 7.0. EC_50_ values were determined using the following equation.

$${\text{y}} = {1}00{ \times }{1}/\left[ {{1} + \left( {{\text{EC}}_{{{5}0}}^{{{\text{Hill}}\;{\text{slope}}}} /{\text{x}}^{{{\text{Hill}}\;{\text{slope}}}} } \right)} \right]$$

### Protein crystallization and X-ray diffraction experiments

The purified enzyme was concentrated to 200 µM and incubated on the ice at a protein:inhibitor stoichiometric ratio of 1:10. The hanging drop vapor diffusion method was used to set up crystal screens by mixing equal volumes of protein-inhibitor sample and reservoir solution. Crystallization conditions for the HD2, HD3, TGX221, and Imatinib complexes were 0.2 M potassium phosphate dibasic and 20% w/v polyethylene glycol 3,350. Crystallization conditions for the HD6 complex were 0.2 M sodium sulfate and 20% w/v polyethylene glycol 1,000. Crystallization conditions for the SGH complex were 0.2 M succinic acid pH 7.0 and 20% w/v polyethylene glycol 1,000. After overnight incubation at 16 °C, needle-shaped single crystals were obtained. For each complex, crystals were cryoprotected by the addition of glycerol directly to the drop to obtain a final concentration of 10% v/v, immediately harvested from the drops, and flash-cooled in liquid nitrogen for X-ray diffraction experiments. X-ray diffraction data were collected at 100 K on beamline D, LS-CAT at the Advanced Photon Source (APS) in Argonne National Laboratory. The diffraction data were indexed, integrated, and scaled using DIALS (Diffraction Integration for Advanced Light Sources)^14^. The phase solutions were obtained from molecular replacement with Phaser using a previously solved *M. tb* EgtD structure as the search model^15^. After a round of rigid-body refinement and simulated annealing, restraints for each ligand were generated using eLBOW (electronic Ligand Building and Optimization Workbench) and manually fitted to the difference density using Coot^16^. The models were subjected to rounds of XYZ coordinate, real-space, occupancy, and B-factor refinement in Phenix refine between manual building in Coot^17,18^.
